# Effects of an incremental theory of personality intervention on the reciprocity between bullying and cyberbullying victimization and perpetration in adolescents

**DOI:** 10.1371/journal.pone.0224755

**Published:** 2019-11-15

**Authors:** Esther Calvete, Izaskun Orue, Liria Fernández-González, Angel Prieto-Fidalgo

**Affiliations:** University of Deusto, Bilbao, Spain; University of Oviedo, SPAIN

## Abstract

The incremental theory of personality interventions (ITPI) teaches adolescents that people can change. Researchers have found that these interventions can reduce the perpetration of bullying and cyberbullying. Moreover, there is reciprocity between perpetrating bullying behaviors and being a victim of them. The objective of this study was to examine whether the ITPI reduces the reciprocity between victimization and perpetration of bullying and cyberbullying. A sample of 858 high school students (52% boys) aged 12 to 17 at pretest (*M* = 14.56, *SD* = 0.97) participated in a double-blind randomized controlled trial (452 participants were assigned to the experimental condition and 406 to the control condition). Measures of bullying and cyberbullying were taken at baseline, six-month, and 12-month follow-ups. The results indicated that victimization was a strong predictor of perpetration for bullying and cyberbullying over time. Perpetration was not a predictor of victimization. Consistently, for both forms of aggressive behavior, the intervention reduced the intensity of the association between victimization and perpetration. This effect was not moderated by the age or sex of the participants. Finally, the effectiveness of the ITPI was moderated by age. Specifically, among the youngest (< 14.48 years), those who received the ITPI showed a slight tendency to reduce aggressive behavior that contrasted with the growing trend in the control group. Among the oldest participants (> 14.48), the trajectories were similar in the two groups. Our findings show that influencing adolescents’ reactions to peer aggression victimization is one of the mechanisms that could explain the beneficial effects of the ITPI and other preventive interventions.

## Introduction

Bullying is a relevant problem widespread in schools. Bullying involves a power imbalance between a target and his or her perpetrator(s) and tends to be repeated over time [1]. Reports indicate that the bullying perpetration rates range between 6.4% and 11% [2,3], and victimization rates range between 21% and 48.5% [2–5]. The consequences for victims can be dramatic because peer victimization is associated with numerous mental health problems and a considerable decline in quality of life [6,7]. In recent years, bullying has spread to social networks. In this context, cyberbullying has been described as willful and repeated harm inflicted [onto a victim] through the use of computers, cell phones, and other electronic devices [8]. Cyberbullying rates are also high, oscillating between 17% and 24% for perpetration [9,10] and between 24% and 26.6% for victimization [9,10].

The perpetration and victimization of peer aggressions tend to overlap [11]. Thus, the percentages of pure victims and pure perpetrators were found to be relatively lower than those known as bully-victims and cyberbully-victims [12]. An attempt was made to explain the existence of this double role through mechanisms of reaction to aggression. Those children and adolescents who are targets of aggression by other youths might seek revenge and act aggressively against their assailants. Similarly, when a child or adolescent bullies others, she/he can—as a result—become the target of bullying and cyberbullying behaviors [13]. In support of these reciprocal mechanisms, some studies have found longitudinal evidence of reciprocity between victimization and perpetration [14]; however, other studies have found only evidence for victimization as a predictor of perpetration. For example, in a longitudinal study, traditional bullying victimization predicted traditional bullying perpetration and cybervictimization predicted cyberbullying perpetration six months later, but perpetration did not predict the increase in victimization [15]. Similarly, in a longitudinal study of traditional bullying, victimization increased the likelihood of involvement in bullying perpetration [16]. In another study, cybervictimization predicted cyber perpetration in girls but not in boys, and perpetration did not predict victimization in girls or boys [17]. In a longitudinal study of three waves, cyber victimization predicted an increase in cyber perpetration through the mediation of cyber witnessing [18]. Other studies have found evidence for the opposite relationship. For example, in a large sample in South Korea, adolescents who bullied others were highly likely to be bullied by others in the following year [19]. Finally, another study did not find bidirectional longitudinal associations over time between cyber victimization and perpetration [20].

The severe consequences of bullying and cyberbullying have encouraged the development of numerous preventive intervention programs in the school setting (for a review see [21–24]). The results of these interventions have been mixed, especially regarding cyberbullying [22]. For example, in a review by Evans and collaborators [23], they found that only 11 of the 22 reviewed studies (50%) displayed significant effects. Furthermore, several review studies have found that the effectiveness of interventions may be moderated by the degree of development of children and adolescents (for example, [25,26]). Although the findings are contradictory, some reviews concluded that the effects of interventions decrease in older youth. For instance, through a hierarchical meta-analysis, [26] found that traditional antibullying interventions were effective from early childhood to early adolescence and that there was a decline to a null effect in the case of interventions with middle adolescents (Grade 8 and higher). The same finding has been observed in investigations of cyberbullying prevention interventions. For instance, in an examination of the effects of the KiVa antibullying program on cyberbullying and cybervictimization, the effect of KiVa on cyberbullying was moderated by age; thus, the reduced odds of endorsing higher frequencies of cyberbullying in the posttest in the experimental condition were observed only when students’ age was below the sample mean [27].

A suggested explanation for the aforementioned findings is that whereas younger youth may be willing to accept the authority of adults and follow curriculum activities [28], older youth may be especially motivated to be respected and maintain their autonomy with regard to adults [29]. For this reason, older adolescents may be especially sensitive to attempts by adults to modify their behavior through interventions and react in an opposite manner than expected. Notably, some interventions with individuals in middle and late adolescence have produced an opposite result of the intended effect [30].

A new approach to interventions, termed “wise interventions,” is attracting interest (for a review see [31]). Wise interventions emphasize the subjective creation of meanings, how people interpret themselves and social situations, and, in doing so, can effectively change behavior in a recursive manner over time [32]. A proliferation of wise interventions has addressed several social and personal problems. Many of these interventions have shocking results because they tend to be very brief in duration and produce lasting changes in people's behavior. Wise interventions do not address a lack of capacity or risk behaviors directly and incorporate three basic motives that drive the search for meaning: (a) precision (people want their interpretations to be accurate); (b) self-integrity (people want to think about themselves as adequate, moral, and competent); (c) belonging (people want to feel accepted and included by others, belong to social groups, and contribute positively to the lives of others).

Wise interventions allow people to adopt new belief or behavior themselves rather than follow a directive from someone else (for example, [33,34]). In this manner, the design of wise interventions allows them to be perceived as respectful regarding the autonomy and status of students so that students realize that they make their own decisions [29]. The results obtained with this type of intervention in adolescent behaviors are enormously promising. For example, Yeager and his collaborators [35,36] designed a short, universal wise intervention designed to change the implicit beliefs about the personality of adolescents (Incremental Theory of Personality Intervention; ITPI). Specifically, the intervention focused on teaching that personality can change. The key elements of the ITPI were as follows: (a) students have an active role to facilitate the deeper processing of the message; (b) the intervention is not presented to the students to change their behavior so that the students do not feel manipulated, and this reduces their resistance to the intervention; and (c) the intervention has long-term effects due to the recursive processes that influence the effects that accumulate over time. The ITPI has been shown to reduce the symptoms of anxiety and depression among adolescents (for example, [35,37–39]) and bullying behavior [35,40]. The results obtained show that their effectiveness is moderated by the level of development of adolescents. For example, in a sample of adolescents, the effect of the intervention on the reduction of cyberbullying perpetration was greater when the level of testosterone, an indicator of pubertal maturation, was lower [40].

Researchers have not examined whether bullying and cyberbullying prevention interventions affect the aforementioned reciprocity between victimization and perpetration. As a result of the intervention, adolescents could learn to not react aggressively when they are the target of rejection and aggression by peers, breaking the vicious circle of violence. This learning could occur because of interventions based on incremental theories of personality. The ITPI teaches that when young people behave aggressively or reject peers they often do so because they have personal problems or do not know how to behave differently and that aggressions do not last forever because people can change. Therefore, this type of intervention could affect adolescents’ reaction when they are victims of aggression, reducing the likelihood of reacting aggressively. Notably, Yeager and colleagues found that following an ambiguous provocation scenario, the ITPI reduced hostile attributions, vengeful desires, and aggressive retaliation [36,39].

### This study

Two studies have found that an ITPI reduced aggressive behavior in adolescents [36,40]. However, additional knowledge is required to understand the mechanisms through which ITPI acts. Although findings have been inconsistent, research has suggested high reciprocity between victimization and perpetration [15,17]. Therefore, in this study, we proposed that the ITPI intervention could reduce the predictive reciprocal associations between victimization and perpetration. This mechanism could partially explain the effectiveness of the intervention. Although in this study we examined victimization and perpetration as outcomes, we expected that the effects of the intervention would be stronger for perpetration because it refers to the behavior of the adolescent receiving the intervention, whereas victimization depends mainly on others’ behavior. Similarly, based on the same reason, we expected that the intervention would reduce the predictive path from victimization to perpetration rather than the path from perpetration to victimization.

Because reviews have indicated that the level of development of the participants can moderate the effects of interventions [29], we included age as a moderator in the analyses. We also controlled for the overall level of perpetration and victimization in the classrooms because the classroom context can influence the behavior of adolescents [41]. Finally, sex was added to the analyses because researchers have found sex differences in the prevalence rates of bullying and cyberbullying victimization and perpetration (for example, [42,43]).

## Materials and methods

### Participants

**Fig 1:**
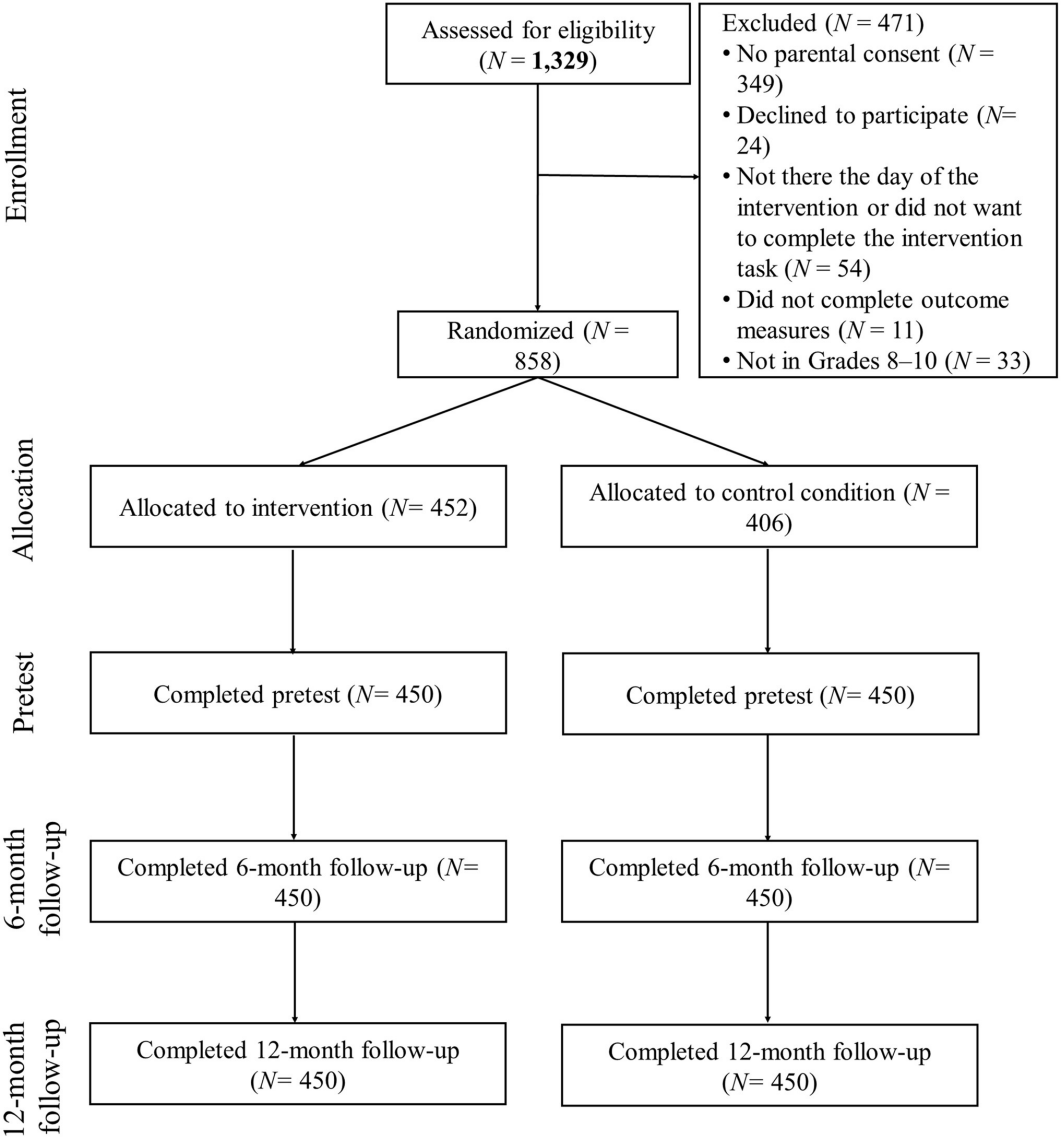
CONSORT diagram.

This study is part of a project conducted in Bizkaia (Basque Country, Spain). Twenty high schools were contacted and 10 agreed to participate. Eligible schools had general student populations, and the schools’ principals agreed to the randomization and delivery of the assigned intervention. Classrooms were selected in each school in order to obtain participants from different grades. The total sample was 1329 adolescents from 33 classrooms. A subsample of 535 adolescents participated in a previous study to assess the effectiveness of the ITPI in bullying and cyberbullying behavior [37]. The total sample was used to test the hypotheses of the current study. Of the total sample, 462 participants were excluded (exclusion criteria are indicated in Fig 1); thus, the final analytic sample was 858 high school students (52% boys) aged 12 to 17 at pretest (*M* = 14.56, *SD* = 0.97). Their parents’ socioeconomic status was determined by following the recommendations of the Working Group of the Spanish Society of Epidemiology and the Spanish Society of Family and Community Medicine [44], who advise researchers to consider parents’ most recent job. This criterion led to the following distribution of socioeconomic class: 15.8% low, 20.8% low-medium, 20.2% medium, 23.8% high-medium, and 19.4% high. The distribution by grade was 280 in 8^th^ grade (*M*_age_ = 13.56, *SD* = 0.52), 363 in 9^th^ grade (*M*_age_ = 14.60, *SD* = 0.47), and 215 in 10^th^ grade (*M*_age_ = 15.80, *SD* = 0.47).

A double-blind randomized controlled trial was performed with two parallel groups. Randomization was performed at the individual level within each classroom on the day of the intervention, and after the pretest, 452 participants were assigned to the experimental condition and 406 to the control condition. Randomization was performed by block and divided by sex, and no differences were observed between the control and the experimental group in the pretest in sex (p = .416) or age (p = .725). The notebooks with the task for each intervention were inside an envelope to ensure blinding of the assignment and condition. The participants completed the intervention individually, and the researchers collected the notebooks inside the envelopes.

Fig 1 displays the participants’ flow chart. No statistically significant differences were found in pretest measures of bullying perpetration, cyberbullying perpetration, and cyberbullying victimization among adolescents who completed all the study measures and those who did not. However, adolescents who did not complete the three study measures were mostly boys (27.8% of boys and 19.7% of girls), χ^2^ (1, *N* = 858) = 7.81, *p* < .01; of older ages (14.79 for noncompleters versus mean age of 14.49 for completers), *t*(856) = 3.86, *p* < .001; and with higher mean scores on bullying victimization (*M* = 3.30 for noncompleters versus 2.61 for completers), *t*(846) = 2.12, *p* < .05.

### Procedure

Students completed the intervention and assessment in their classrooms during school hours. The session was administered by a trained psychology research assistant who led students to work quietly and privately. Informed consent was required both from parents and adolescents. An overview of the procedure is presented in Fig 1. One week before the interventions began, a survey was administered during school hours (pretest), and a similar posttest was administered six months and 12 months afterward (clinical trials ID: NCT03481699). As the majority of the adolescent population in the Basque Country is bilingual, the questionnaires were presented in both Spanish and Basque so that each adolescent could choose the language in which to respond. The Ethics Committee of University of Deusto approved this study.

### Intervention

In this study we delivered an experimental intervention modeled on the intervention developed by Yeager and collaborators [45]. The experimental and control interventions were adapted to local cultural specifications and translated into Spanish and Basque. The experimental intervention was divided in three parts and its total duration was 50–60 minutes. In the first part, participants read a scientific article that provides information about individuals´ potential to change. They read conclusions of neurological and behavioral studies that demonstrate that the thoughts and feelings in the brain control the behavior and that under correct circumstances the pathways in the brain can change. Next, participants wrote three sentences based on the science explaining that people can change. In the second part, participants read extracts written by other students that had participated in the study and written their own conclusions. The purpose of using of the testimonials obtained from other participants was to add credibility to the incremental theory of personality. In the final part, participants described a time when they felt isolated, rejected, or disappointed by another person at school. Next, they imagined that the same event happened to another student and write one to three paragraphs describing what they could do or say to help the other student to understand that people can change and the things happening to him/her might also change.

The control intervention was developed to be performed in parallel with the experimental intervention and also has three main parts (see Supplementary Information, Annex 1 by Yeager and collaborators [46]). However, the control intervention contained scientific information and information on the human brain. In the first part, the participants read a scientific article about the brain, including brain localization and the role of different brain areas in supporting cognitive functioning. In the second part, the participants read stories written by other adolescents explaining how they became accustomed to the sensory and physical environment (e.g., the building, sounds, smells) of high school. In the last part, participants wrote about how and why students adapt to the physical environment at high school.

### Instruments

The Revised Peer Experiences Questionnaire (RPEQ; [47]) was used to assess participants´ bullying perpetration and victimization experiences. The bullying perpetration and victimization scales comprised nine parallel items that asked participants to rate how often they have perpetrated or they have received an aggressive behavior in the last six months on a 5-point scale ranging from 1 (*never*) to 5 (*a few times a week*). Sample items are “I threatened to hurt or beat a peer up” (perpetration scale) and “A kid gossiped about me so that others would not like me” (victimization scale). Researchers that have examined the RPEQ found adequate psychometric properties in studies involving adolescents [38]. Test–retest reliability and internal consistency were adequate [48]. The version of the RPEQ used in this study had been translated into Spanish and Basque by means of back-translation procedures and validated in other studies with good psychometric properties [49,50]. Cronbach coefficients were .81, .87, and .90 at T1, T2, and T3 for perpetration, respectively; and .82, .87, and .87 at T1, T2, and T3 for victimization, respectively.

Cyberbullying victimization and perpetration were assessed using the Cyberbullying Questionnaire (CBQ) [51] to measure perpetration and victimization in the past six months. Each scale includes nine items, for example, “sending pictures of an acquaintance that could be humiliating” (perpetration) and “receiving threatening or insulting messages from other people” (victimization). The following response scale was used: 0 (*never*), 1 (*1 or 2 times*), 2 (*3 or 4 times*), and 3 (*5 or more times*). The CBQ has shown adequate factorial and convergent validity, in addition to an acceptable internal consistency [51,52]. In this study, Cronbach coefficients were .79, .89, and .91 at T1, T2, and T3 for perpetration, respectively; and .74, .84, and .89 at T1, T2, and T3 for victimization, respectively.

### Overview of the statistical approach

The pattern of missingness was examined. In the general sample, for self-reported measures, Little’s MCAR test was statistically significant, χ^2^(235) = 420, *p* < .001. Thus, we used full information maximum likelihood (FIML), a recommended method to manage missing values when they are not distributed randomly. FIML estimates parameters by using all the available data, including cases without data [53]. We used hierarchical linear modeling 7–3 [54] with robust standard errors. We estimated separate models for bullying and cyberbullying victimization and perpetration. Each model was accompanied by Level 1, 2, and 3 equations. For instance, for cyberbullying perpetration, for Level 1, regression equations modeled variation in the repeated measures as a function of time (i.e., the three waves of data) and cyberbullying victimization. Because cyberbullying was measured at each time point, it was modeled as a time-varying covariate to investigate whether cyberbullying perpetration was predicted by changes in cyberbullying victimization over time. Time was coded as 0, 1, or 2.

For Level 2, the equations modeled individual differences in the Level 1 parameters (i.e., intercepts and slopes) as a function of between-subject variables. Level 2 predictors of the intercept included condition (0 = control, 1 = experimental) and age. Level 2 predictors of the slope included the same predictors and interaction terms between the condition and the other variables. The inclusion of these parameters at Level 2 allowed us to test the effects of condition and age on both the intercept and the change in the outcome variables over time. Sex (1 = female, 0 = male) was also included as a predictor of the intercept to control for sex differences in cyberbullying perpetration. We included random effects for intercept and time at Level 2, allowing for variability between individuals in the initial levels and changes over time. Where the random effects were not significant, they were removed from the final models. We tested models that included sex and the Sex x Time, Sex x Condition, and Sex x Condition x Time interaction terms. Sex was a significant predictor of the intercept. However, because sex did not moderate the effect of the intervention on victimization nor on perpetration, we report the most parsimonious models without these components.

Finally, at Level 3, we included the classrooms and their average level of cyberbullying perpetration as a predictor of the intercept and the time slope. Thus, the level of cyberbullying in each classroom focused on the grand mean. Random effects for intercept and slope were included, allowing variation between classrooms. A similar model was used to predict bullying perpetration. In this case, bullying victimization was used as a predictor, instead of cyberbullying victimization, and the average level of bullying perpetration in the classroom was used at Level 3. We also tested parallel models in which the outcome was cyberbullying versus bullying victimization and predictors were cyberbullying versus bullying perpetration. For these models, the average level of victimization in the classroom was used at Level 3.

The effect sizes were calculated to compare the differences between groups in changes in the outcome variables from baseline to follow-up using the estimated marginal means obtained in the mixed models. Where the differences between groups in changes were statistically significant, positive values of Cohen’s *d* indicated greater decreases in outcomes (e.g., greater reduction in bullying perpetration) for adolescents in the ITPI group compared with the adolescents in the control condition. Negative values of Cohen’s *d* indicate greater decreases in the control group. Data is available at https://osf.io/mh92w/ (DOI 10.17605/OSF.IO/MH92W).

## Results

### Descriptive statistics and preliminary analyses

Table 1 shows the correlation coefficients for the study variables. All the variables were significantly associated (*p* < .001). The highest correlations were between perpetration and victimization at each time for traditional bullying and cyberbullying. Table 2 depicts the descriptive statistics and comparisons between the intervention and control conditions. Several *t* tests indicated no significant differences between the control and ITPI groups in any study variable at baseline (*p* > .05). There were not significant differences in any variable depending on socioeconomic level, except for cyberbullying perpetration at the one-year follow-up, in which adolescents of medium socio-economic class scored lower than adolescents of low-medium (*p* = .007) and high-medium (*p* = .005) socioeconomic classes.

**Table 1:** Correlation coefficients between variables.

	1	2	3	4	5	6	7	8	9	10	11
1.T1 BP											
2.T2 BP	.33*										
3.T3 BP	.26*	.42*									
4.T1 BV	.52*	.21*	.20*								
5.T2 BV	.28*	.66*	.34*	.48*							
6.T3 BV	.21*	.34*	.68*	.29*	.41*						
7.T1 CBP	.59*	.30*	.19*	.45*	.29*	.15*					
8.T2 CBP	.32*	.78*	.35*	.22*	.65*	.27*	.40*				
9.T3 CBP	.30*	.34*	.67*	.20*	.28*	.59*	.27*	.33*			
10.T1 CBV	.46*	.20*	.22*	.60*	.31*	.26*	.59*	.26*	.28*		
11.T2 CBV	.29*	.59*	.36*	.36*	.68*	.38*	.34*	.77*	.39*	.36*	
12.T3 CBV	.30*	.28*	.62*	.25*	.32*	.71*	.27*	.32*	.66*	.33*	.42*

*Note*. T1 = pretest; T2 = 6-month follow-up; T3 = 12-month follow-up; B = bullying; CB = cyberbullying; P = perpetration; V = victimization.

* *p* < .001.

**Table 2:** Descriptive statistics and comparisons between the intervention and control conditions.

	Total	Intervention	Control	*t*	*p*
	*M*(*SD*)	*M*(*SD*)	*M*(*SD*)		
T1 BP	1.96 (3.20)	2.01 (3.28)	1.91 (3.10)	0.45	.651
T2 BP	1.92 (3.68)	1.81 (3.47)	2.03 (3.88)	-0.81	.421
T3 BP	1.88 (4.11)	1.85 (4.31)	1.92 (3.87)	-0.21	.830
T1 BV	2.77 (4.01)	2.94 (4.21)	2.59 (3.75)	1.27	.206
T2 BV	2.46 (4.21)	2.48 (4.35)	2.45 (4.06)	0.10	.924
T3 BV	2.25 (4.02)	2.33 (4.30)	2.17 (3.68)	0.51	.690
T1 CBP	1.12 (2.18)	1.21 (2.35)	1.01 (1.98)	1.35	.178
T2 CBP	1.21 (3.04)	1.13 (2.86)	1.31 (3.22)	-0.82	.413
T3 CBP	1.11 (3.15)	1.09 (3.11)	1.13 (3.20)	-0.17	.862
T1 CBV	1.38 (2.43)	1.42 (2.45)	1.34 (2.40)	0.53	.596
T2 CBV	1.30 (2.85)	1.22 (2.81)	1.39 (2.88)	-0.83	.406
T3 CBV	1.25 (3.06)	1.26 (3.25)	1.24 (2.84)	0.09	.929

*Note*. T1 = pretest; T2 = 6-month follow-up; T3 = 12-month follow-up; B = bullying; CB = cyberbullying; P = perpetration; V = victimization.

* *p* < .05,

**p < .01,

***p < .001

### Effects of the ITPI

Table 3 presents the results of the mixed models for bullying and cyberbullying perpetration as outcomes, and Table 4 presents the random effects. The average level of bullying/cyberbullying perpetration in the classroom was significantly associated with the intercept; thus, the classroom context is a predictor of the initial individual level. Sex was significantly associated with initial level of perpetration with girls scoring lower than boys in both bullying and cyberbullying perpetration. There were no differences in initial levels of perpetration between the experimental and control groups. Although the slope for time was not significant, it indicated an increasing tendency over time for both bullying and cyberbullying perpetration. For bullying but not for cyberbullying, the level of perpetration in the classroom predicted a lower increasing tendency over time, probably because classrooms with high levels of aggressive behavior have less space to increase aggressive behavior. The negative significant time x age interaction indicates that bullying and cyberbullying increase less as age increases. Moreover, as hypothesized, the time x condition x age interaction was statistically significant, indicating that the effect of the intervention on perpetration levels is moderated by age.

**Table 3:** Results of mixed linear models predicting intervention effects on the bullying/cyberbullying perpetration trajectories over time (fixed effects).

Predictors of bullying	Coefficient	SE	*t*	*p*	Predictors of cyberbullying	Coefficient	SE	*t*	*p*
Intercept	2.26	0.18	12.49	< .001	Intercept	1.37	0.12	11.69	< .001
Average bullying perpetration in the classroom	0.71	0.07	9.48	< .001	Average cyberbullying perpetration in the classroom	0.71	0.09	7.52	< .001
Condition	0.05	0.22	0.25	.806	Condition	0.18	0.13	1.40	.806
Sex (1 = female)	-0.98	0.19	-5.13	< .001	Sex (1 = female)	-0.72	0.14	-5.18	< .001
Age	0.04	0.07	0.57	.566	Age	0.01	0.04	0.24	.813
Bullying victimization	0.61	0.06	10.63	< .001	Cyberbullying victimization	0.70	0.07	10.15	< .001
Condition x Bullying victimization	-0.16	0.07	-2.29	.023	Condition x Cyberbullying victimization	-0.25	0.09	-2.75	.006
Age x Bullying victimization	0.04	0.06	0.66	.510	Age x Cyberbullying victimization	-0.07	0.09	-0.81	.418
Condition x Age x Bullying victimization	-0.04	0.09	-0.51	.610	Condition x Age x Cyberbullying victimization	-0.01	0.13	-0.04	.993
Time	0.12	0.09	1.34	.187	Time	0.07	0.08	0.92	.365
Time x Average bullying perpetration in the classroom	-0.18	0.09	-2.10	.042	Time x Average cyberbullying perpetration in the classroom	-0.13	0.10	-1.34	.188
Time x Condition	-0.05	0.13	-0.37	.708	Time x Condition	-0.11	0.09	-1.27	.208
Time x Age	-0.27	0.10	-2.81	.005	Time x Age	-0.21	0.08	-2.73	.007
Time x Condition x Age	0.32	0.14	2.22	.027	Time x Condition x Age	0.22	0.09	2.30	.022

**Table 4:** Final estimation variance components.

Random Effect	SD	Variance Component	*df*	χ^2^	*p*
Cyberbullying perpetration					
Level 2 intercept	1.55	2.39	732	1485	< .001
Level 2 time slope	0.61	0.37	732	901	< .001
Level 3 time slope	0.23	0.05	42	71	.004
Bullying perpetration					
Level 2 intercept	1.87	3.51	773	1371	< .001
Level 2 time slope	0.78	0.60	730	905	< .001
Level 3 time slope	0.27	0.07	42	66	.011
Cyberbullying victimization					
Level 2 intercept	1.76	3.11	775	1757	< .001
Level 2 time slope	0.67	0.44	732	963	< .001
Bullying victimization					
Level 2 intercept	3.16	10.00	773	2299	< .001
Level 2 time slope	0.89	0.78	730	928	< .001
Level 3 time slope	0.24	0.24	42	61	.028

Note. Only statistically significant components are displayed.

**Fig 2:**
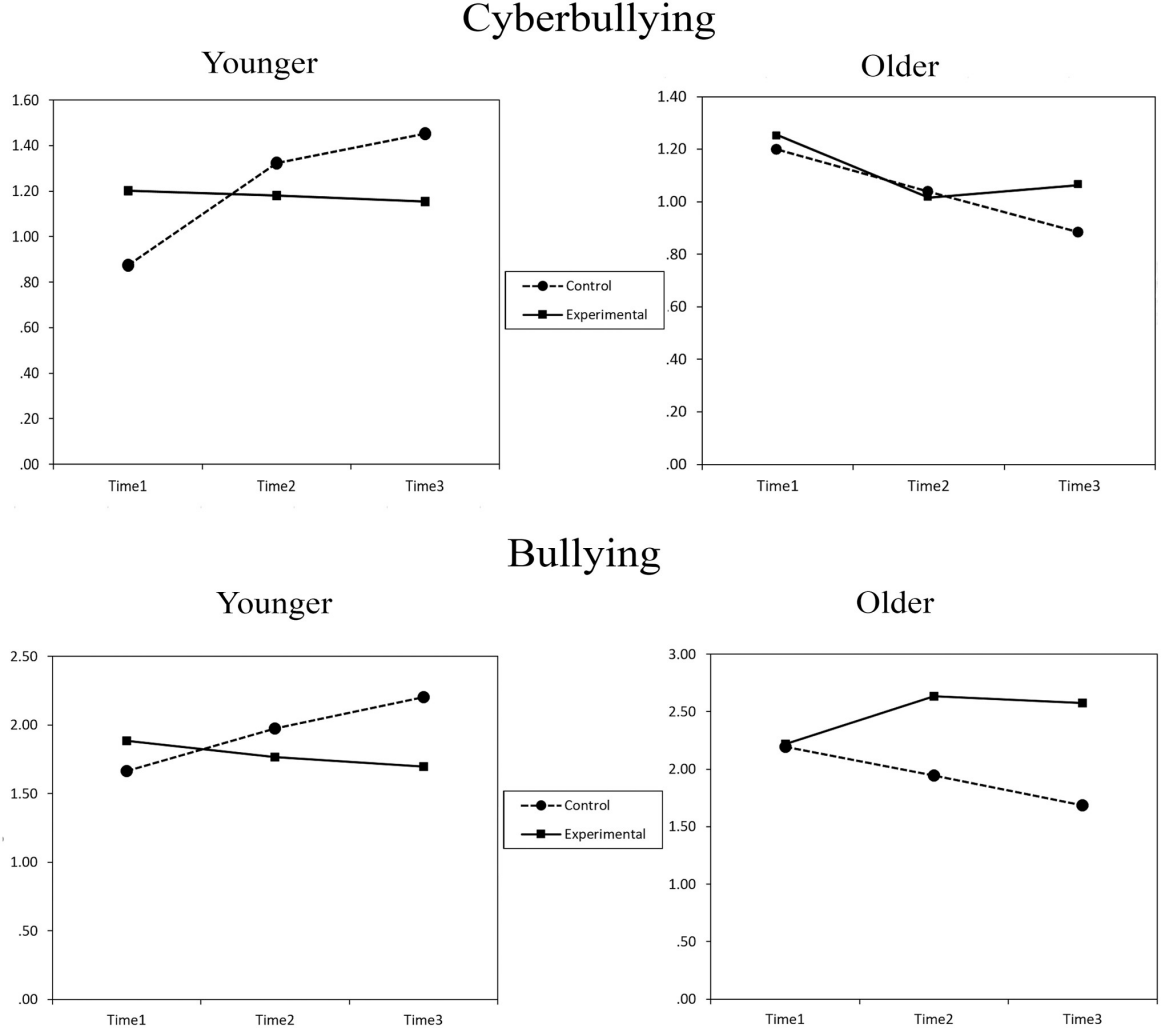
Trajectories of bullying and cyberbullying perpetration in control and experimental groups by age.

Fig 2 displays the trajectories of both bullying and cyberbullying perpetration for adolescents within the experimental and control conditions of participants under and above the median age (Median = 14.48). For cyberbullying, among younger participants, those in the experimental group display a slightly decreasing trend, whereas those in the control group display an increasing tendency. Among older participants, the trajectories are very similar in the experimental and control groups. For bullying, a similar pattern is observed among the younger participants. In the case of older participants, the experimental group displays an increasing tendency from the pretest to the six-month follow-up and a slight tendency to decrease from the six-month to the one-year follow-up. The control group, by contrast, displays a stably decreasing trend from the pretest to the one-year follow-up. Table 5 presents the means for bullying and cyberbullying perpetration over time estimated in the mixed models. The effect sizes comparing mean change scores from baseline to 6-month follow-up, and from baseline to the 12-month follow-up, were small.

**Table 5:** Estimated marginal means and standard errors (in parenthesis) of the mean (calculated from mixed linear effects models) for bullying and cyberbullying perpetration.

	ITPI			Control			Cohen’s d based on mean change score [95% CI]	
Bullying	Baseline	6 months	12 months	Baseline	6 months	12 months	Baseline to 6 months	Baseline to 12 months
Younger	1.89 (0.17)	1.77 (0.18)	1.70 (0.19)	1.67 (0.18)	1.98 (0.19)	2.20 (0.20)	**0. 20** [-0.01, 0.40]	**0.26** [0.04, 0.47]
Older	2.04 (0.17)	1.89 (0.19)	1.99 (0.19)	2.20 (0.77)	1.94 (0.19)	1.69 (0.20)	**-0.05** [-0.25, 0.16]	**-0.18** [-0.39, 0.04]
Cyberbullying	Baseline	6 months	12 months	Baseline	6 months	12 months	Baseline to 6 months	Baseline to 12 months
Younger	1.20 (0.13)	1.18 (0.14)	1.15 (0.14)	0.88 (0.14)	1.32 (0.15)	1.45 (0.15)	**0. 22** [0.04, 0.45]	**0.27** [0.07, 0,49]
Older	1.25 (0.13)	1.02 (0.14)	1.06 (0.15)	1.20 (0.14)	1.04 (0.15)	0.88 (0.16)	**-0.02** [-0.18, 0.23]	**-0.15** [-0.36, 0.06]

**Fig 3:**
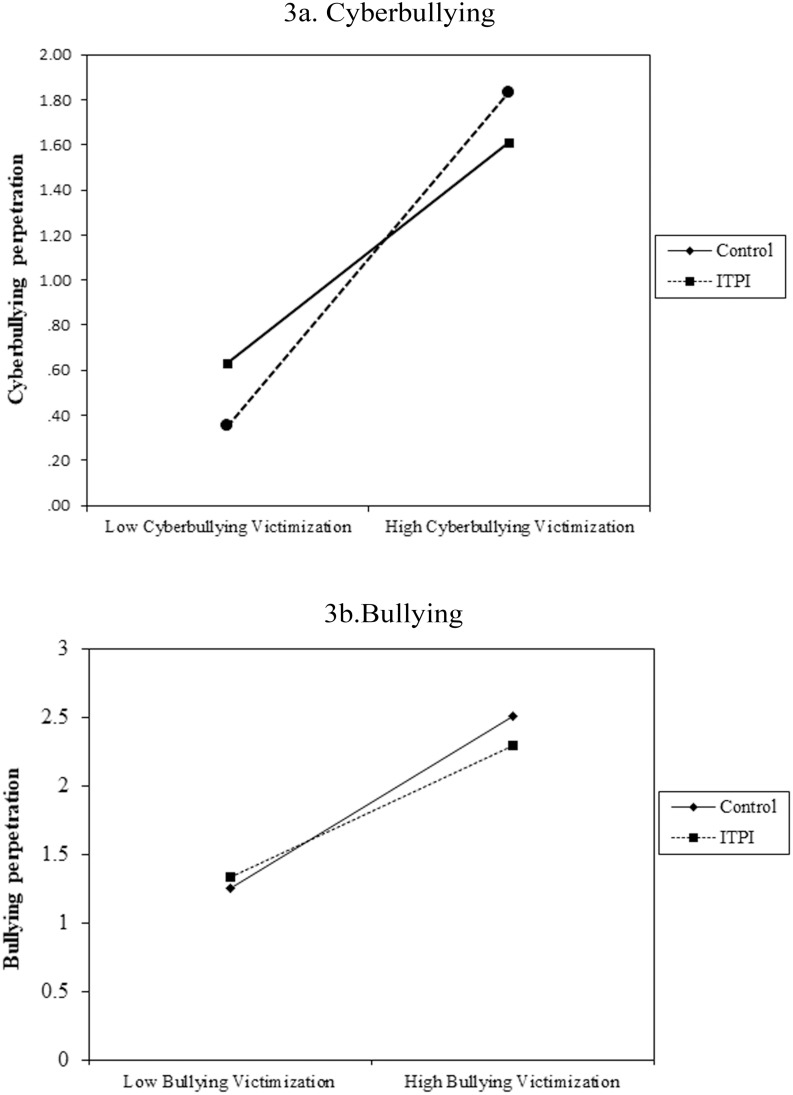
Association between perpetration and victimization depending on the group assignation (experimental-control) in cyberbullying (3a) and bullying (3b).

The results also indicate that victimization was a strong predictor of perpetration for bullying and cyberbullying. Because victimization was included as a Level 1 variable, these results indicate that perpetration increases when victimization increases. More important, the intervention reduced the predictive association between victimization and perpetration. Fig 3 displays this association for adolescents in the experimental and control groups. As observed, the association is more intense in the control group than in the experimental group. Age did not moderate the role of victimization on the prediction of perpetration or the effect of the intervention on the association between victimization and perpetration.

Table 6 presents the results for bullying and cyberbullying victimization as outcomes. Average level of victimization in the classroom was a strong predictor of initial level of individual victimization for bullying and cyberbullying. The changes in both bullying and cyberbullying perpetration were strong predictors of both bullying and cyberbullying victimization over time, respectively. However, no effect of age and condition were observed for victimization.

**Table 6:** Results of mixed linear models predicting intervention effects on the bullying/cyberbullying victimization trajectories over time (fixed effects).

Predictors of bullying victimization	Coefficient	*SE*	*t*	*p*	Predictors of cyberbullying victimization	Coefficient	*SE*	*t*	*p*
Intercept	1.19	0.17	6.82	< .001	Intercept	1.55	0.15	9.99	< .001
Average bullying victimization in the classroom	0.62	0.08	8.06	< .001	Average cyberbullying victimization in the classroom	0.89	0.07	13.15	< .001
Condition	0.34	0.31	1.11	.268	Condition	-0.01	0.15	-0.01	.991
Sex (1 = female)	-0.01	0.17	-0.06	.952	Sex (1 = female)	-0.36	0.16	-2.19	.029
Age	-0.23	0.15	-1.56	.120	Age	-0.08	0.05	-1.64	.102
Bullying perpetration	0.67	0.04	15.1	< .001	Cyberbullying perpetration	0.60	0.07	9.03	< .001
Condition x Bullying perpetration	-0.02	0.08	-0.19	.847	Condition x Cyberbullying perpetration	-0.11	0.11	-0.94	.349
Age x Bullying perpetration	0.04	0.04	1.09	.277	Age x Cyberbullying perpetration	-0.03	0.05	-0.47	.639
Condition x Age x Bullying perpetration	0.01	0.07	0.17	.860	Condition x Age x Cyberbullying perpetration	0.04	0.10	0.39	.696
Time	-0.16	0.09	-1.74	.083	Time	-0.09	0.07	-1.34	.180
Time x Average bullying victimization in the classroom	-0.24	0.07	-3.54	< .001	Time x Average cyberbullying victimization in the classroom	-0.17	0.09	-1.92	.056
Time x Condition	-0.05	0.16	-0.33	.742	Time x Condition	0.05	0.11	0.48	.632
Time x Age	0.07	0.09	0.76	.450	Time x Age	-0.01	0.06	-0.07	.948
Time x Condition x Age	0.05	0.08	0.62	.533	Time x Condition x Age	0.04	0.07	0.48	.630

## Discussion

Peer aggression is a highly prevalent problem among adolescents. In addition, victimization and perpetration tend to overlap [11]; that is, many adolescents react with violence when subjects of aggression because they are motivated by the desire to take revenge [17,55]. The objective of this study was to examine whether an intervention aimed at teaching an incremental theory of personality, that is, that people can change, reduced the reciprocity between victimization and perpetration of bullying and cyberbullying.

We expected that the effects of the intervention would be stronger for perpetration than victimization because perpetration involves the behavior of the adolescent receiving the intervention, whereas victimization depends mainly on others’ behavior. In consistency with our hypothesis, the ITPI had no effect on the trajectory of victimization over time or on the predictive relationship between perpetration and victimization, neither for bullying nor for cyberbullying. This result is not surprising because the victimization does not depend mainly on the behavior of the recipients of the intervention but on the behavior of other people, including people outside of the study. The results suggest that the ITPI acts on the behaviors of the adolescents who receive them.

We also predicted that victimization would act as an antecedent of perpetration and that the intervention would reduce the predictive path from victimization to perpetration. As we expected, and consistent with other studies [14,15,17], victimization was a strong predictor of perpetration over time for bullying and cyberbullying. By contrast, perpetration was not a predictor of victimization. Consistent in both forms of aggressive behavior, the intervention reduced the intensity of the association between victimization and perpetration. This effect was not moderated by the age or sex of the participants. Namely, the findings indicate that the probability of perpetrating bullying/cyberbullying as a consequence of victimization was lower among adolescents who received the ITPI than in adolescents in the control group. This finding is critical because it suggests that the ITPI can contribute to the subjective creation of meanings [32] such that when adolescents experience rejection and aggression from others, they could interpret these experiences in a more benign manner, which inhibit retaliatory reactions. The ITPI teaches through stories of other young people that often adolescents who behave rudely to others or commit acts of aggression do so because they have personal problems or do not know how to behave differently. The ITPI also teaches that people can change. Therefore, when adolescents who have received the ITPI are the target of aggressions, they could interpret that the other person’s actions may be caused some difficulty and that the attacks will likely not last forever; thus, the desire for revenge and the probability of reacting aggressively would be reduced. This is consistent with the results obtained by Yeager and collaborators [36], that is, after the ITPI the adolescents reacted to provocation with a more positive attitude and less desire for revenge.

Although the main objective of the study was not to test the effects of the ITPI on the trajectory of bullying and cyberbullying, the results show that its effectiveness is moderated by age. Specifically, in terms of cyberbullying, among the youngest adolescents (<14.48), those who received the ITPI showed a slight tendency to reduce aggressive behavior that contrasts with the increasing trend in the control group. Among the oldest ones, the trajectories were similar in the two groups. This result largely coincides with that obtained with the KiVa program by Williford and collaborators [27]. In the case of bullying, the result among the youngest is very similar to that obtained for cyberbullying. However, among the oldest participants, there is a paradoxical effect of a greater reduction in the perpetration of bullying in the control group than in the experimental group. This result is reminiscent of the findings obtained with other interventions in which results opposite to those expected have been observed (for a review see [29]). Traditional bullying behavior takes place face to face, and it is possible that among older adolescents, who are more motivated to maintain their social status, even a subtle intervention, such as that used in this study, may provoke opposite reactions.

Finally, male adolescents scored higher than female adolescents on perpetration of bullying and cyberbullying and on cyber victimization. However, sex did not moderate the effects of the ITPI in the outcome trajectories or in the predictive association between victimization and perpetration. Thus, we dropped sex from the predictive models to increase their parsimony, and sex was maintained only as a predictor of the baseline level.

This study has limitations that provide opportunities for further research. One limitation is the exclusive use of self-reports, which can contribute to increasing the association between victimization and perpetration. A second limitation is the loss of participants over time, especially in the one-year follow-up. Some adolescents had changed schools and many others could not be present in the classroom on the days when the data was collected. The oldest boys with higher scores on bullying victimization were the participants with the highest likelihood of not completing all the study measures; thus, the results may not be generalizable to this particular group of participants. Finally, the study is based exclusively on quantitative data; thus, conducting qualitative studies to examine how adolescents create meanings after the intervention would be valuable. Thus, for example, a topic for further research could be an evaluation of how adolescents interpret victimization behaviors and if they do so in a more benign manner than the adolescents in the control group. Despite these limitations, the study has strengths that allow for valuable conclusions. An RCT design was used with a large sample of adolescents, The study included a follow-up of six months and one year. In addition, contributions were made to the field of preventive bullying and cyberbullying intervention by examining how an intervention alters the reactions of adolescents to victimization.

### Conclusions

Findings of this study indicate that a brief intervention teaching that people can change reduces the reciprocity between victimization and perpetration. This mechanism could explain the beneficial effects of the ITPI and of other interventions. The intervention is within the so-called Wise Interventions, and its brevity makes it a low-cost tool easily implemented in educational contexts. The results also indicate that early implementation of the intervention is critical because its effectiveness was greater among the youngest participants.
